# Tracheostomy intervention in intubated COVID positive patients: A survey of current clinical practice among ENT surgeons

**DOI:** 10.1002/hed.26274

**Published:** 2020-06-05

**Authors:** Ayman D'Souza, Ricard Simo, Alwyn D'Souza, Francis Vaz, Andrew Prior, Rahul Kanegaonkar

**Affiliations:** ^1^ Christ Church University of Oxford Oxford UK; ^2^ Department of Otorhinolaryngology Guy's and St Thomas' NHS Foundation Trust London UK; ^3^ Department of Otorhinolaryngology University Hospital Lewisham London UK; ^4^ Department of Head and Neck Surgery University College Hospital London UK; ^5^ Department of Otorhinolaryngology Princess Royal University Hospital Kent UK; ^6^ Institute of Medical Sciences, Medway Campus Canterbury Christ Church University Kent UK

## Abstract

**Introduction:**

The COVID‐19 pandemic has resulted in an unprecedented need for critical care intervention. Prolonged intubation and mechanical ventilation has resulted in the need for tracheostomy in some patients. The purpose of this international survey was to assess optimal timing, technique and outcome for this intervention.

**Methods:**

An online survey was generated. Otorhinolaryngologists from both the United Kingdom and Abroad were polled with regards to their experience of tracheostomy in COVID‐19 positive ventilated patients.

**Results:**

The survey was completed by 50 respondents from 16 nations. The number of ventilated patients totalled 3403, on average 9.7% required a tracheostomy. This was on average performed on day 14 following intubation. The majority of patients were successfully weaned (mean 7.4 days following tracheostomy).

**Conclusion:**

The results of this brief survey suggest that tracheostomy is of benefit in selected patients. There was insufficient data to suggest improved outcomes with either percutaneous vs an open surgical technique.

## 1 INTRODUCTION

The global COVID‐19 pandemic has resulted in unprecedented demands on healthcare services.^1^ As of the April 14th, 2020, the World Health Organization reported a global number of 1 844 863 confirmed cases with 117 021 death.^2^ In the United Kingdom there are currently 88 625 confirmed cases and 11 329 in hospital deaths.

This novel betacoronavirus results in pneumonia that may rapidly progress to a severe acute respiratory syndrome.^3^ In such situations intubation and mechanical ventilation is frequently required which has stretched and regularly exceeded capacity in a number of institution.^4^

A tracheostomy is classically recognized to be of benefit in those requiring prolonged ventilation.^5^ Benefits include weaning and pulmonary toilet in those requiring regular airway suction in the Intensive Care Unit. Tracheostomy has been usually undertaken between day 7 and 10 following intubation,^6^ but the optimal timing for tracheostomy in COVID positive patients remains uncertain.

Tracheostomy may be undertaken either via an open surgical approach or percutaneously, usually at the bedside.^7^ Each approach has its own merits, staffing and equipment requirements.

Recently disseminated ENT UK guidelines have recognized the need for caution when performing a tracheostomy.^8^ Tracheostomy is recognized to be an aerosol‐generating procedure that increases the risk of nosocomial amplification to health care workers and other patients.^9^ Full personal protective equipment (PPE) is mandatory for the surgical team and assisting staff, and practical suggestions have been published to reduce the level of aerosol generated during and following the procedure.^10^ However, there remains little data as to the optimal approach and subsequent outcome in COVID positive ventilated patients.

This global pandemic, initially identified in the Wuhan Province in China, has gradually spread across international borders and has now been identified in 210 countries and territories around the world.^11^ Those units initially exposed have developed a greater understanding of how to best manage COVID‐19 positive patients. Their experience is of benefit to other institutions early in the pandemic cycle not only in terms of planning patient pathways and healthcare resources, but also predicting outcomes.

We therefore conducted this international survey to assess tracheostomy intervention in intubated COVID‐19 positive patients amongst ENT Surgeons. We sought to specifically identify at what point this procedure was undertaken, the outcome following intervention, and possible optimal technique.

## 2 METHODOLOGY

Permission to undertake this survey, considered to be a Service Development Project, was obtained from the Research and Development Department of King's College Hospital NHS Foundation Trust, London.

An online questionnaire was constructed on SurveyMonkey consisting of the following questions:

1. In which country/region are you based?
2. How many ventilated COVID‐19 patients have you had at your hospital?
3. What percentage of intubated patients have required a tracheostomy?
4. On average on what day was the tracheostomy performed (eg, day 7 of intubation)?
5. How long after tracheostomy was the patient weaned off the ventilator?
6. What percentage of patients died despite tracheostomy?
7. Were outcomes better with any specific tracheostomy technique (eg, percutaneous vs surgical)?

An email invitation with a link to the survey was disseminated by three of the authors to European Colleagues known to them as fellow examiners on the European Board Examination in Otorhinolaryngology. Those contacted were also invited to disseminate the invitation to Clinicians who may wish to contribute.

The questionnaire was disseminated on the March 27th, 2020 and data accepted until the April 15th, 2020.

Survey data collected was downloaded and transferred to a Microsoft Office Excel spreadsheet for analysis.

### 2.1 Patient and public involvement

Patients and the public were not involved in the production of this survey or article.

## 3 RESULTS

**FIGURE 1:**
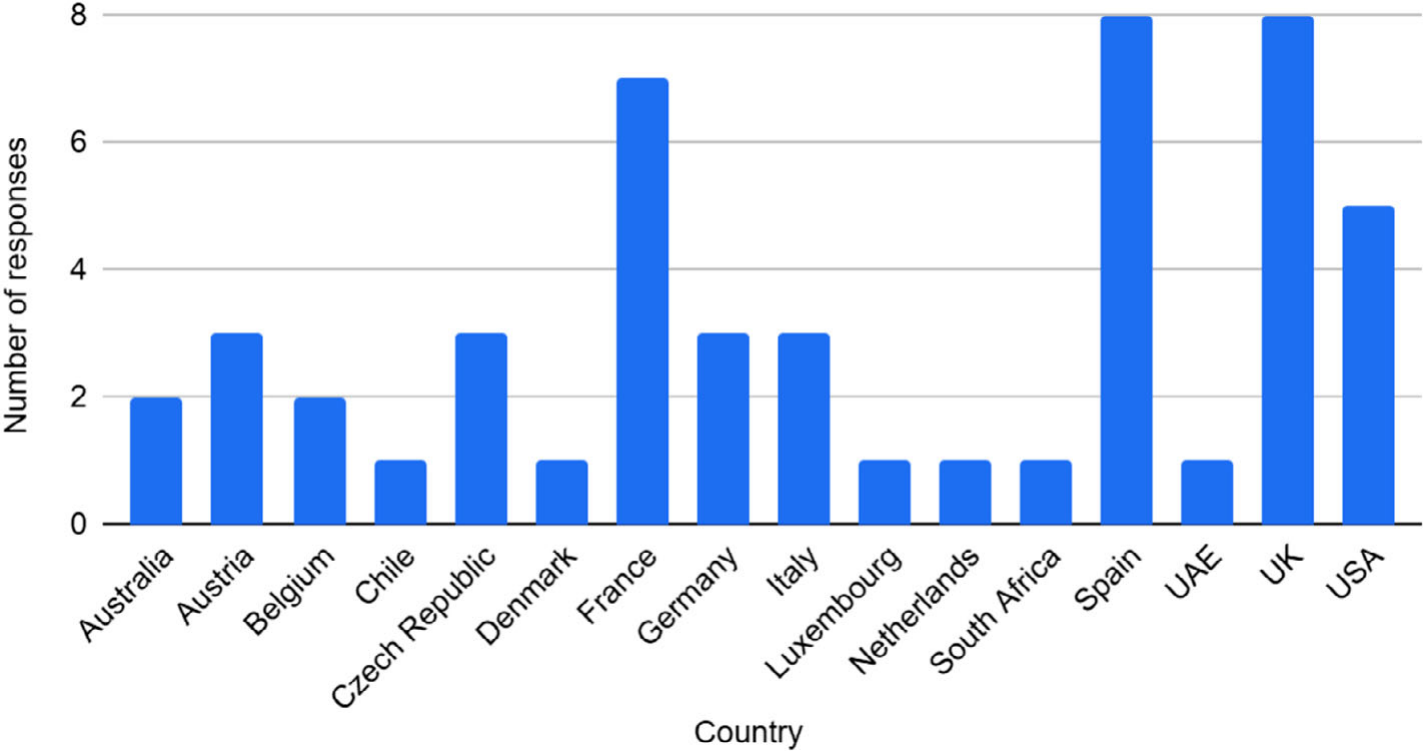
Number of respondents by country [Color figure can be viewed at wileyonlinelibrary.com]

An initial 90 practicing European Consultant Otorhinolaryngologists were contacted. The survey was completed by a total of 50 respondents from both the United Kingdom (n = 8) and International units (Figure 1.)

The number of ventilated patients totalled 3403 with a mean of 68 patients per Hospital Unit/Trust (range 0‐600).

**FIGURE 2:**
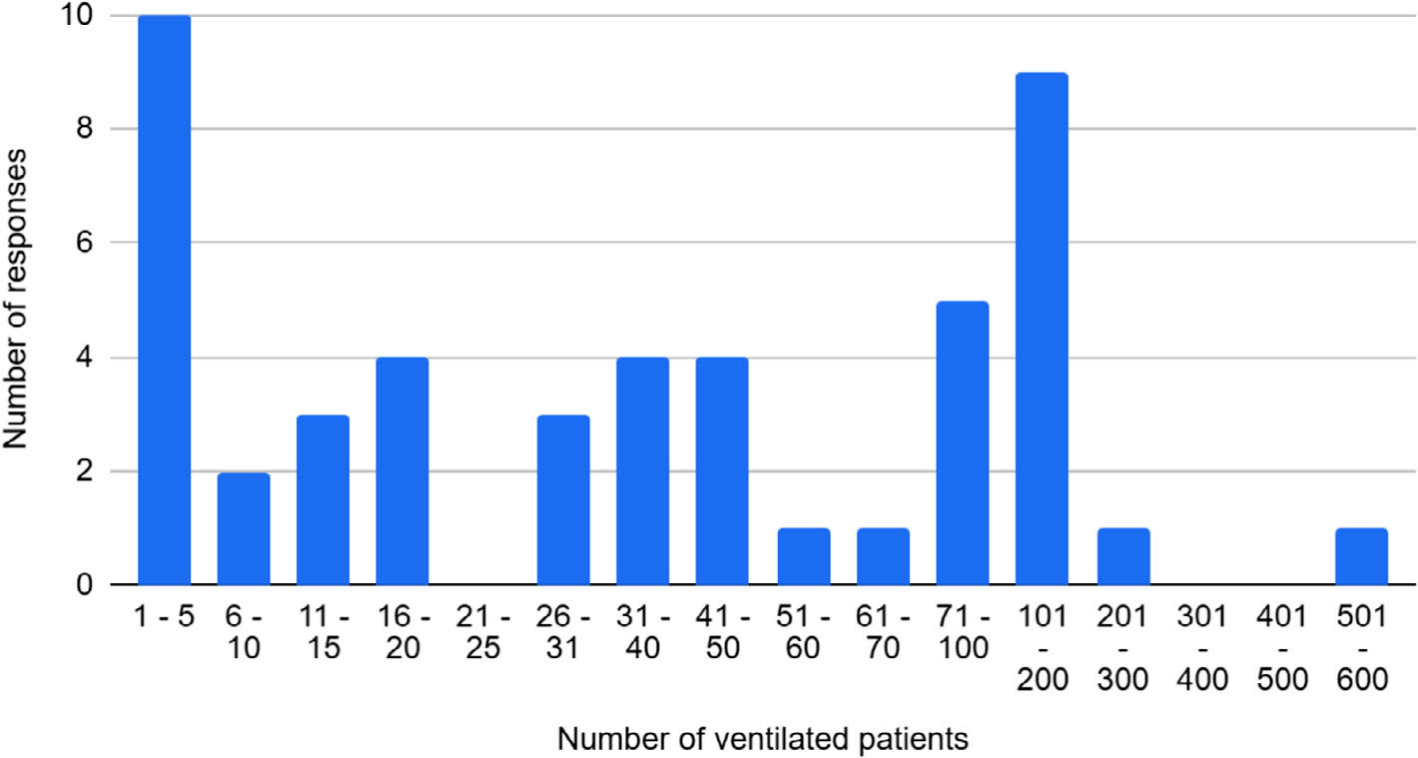
Number of ventilated COVID‐19 patients by Hospital Unit/Trust [Color figure can be viewed at wileyonlinelibrary.com]

The percentage of intubated patients requiring tracheostomy was on average 9.65% (range 0%‐100%) with a tracheostomy performed following intubation at a mean of 14.4 days (range 7‐21). This was drawn from 28 respondents from a pool of 2701 intubated and ventilated patients (Figure 2).

Patients were successfully weaned post tracheostomy on average after 7.4 days (range 1‐16 days). Despite undergoing a tracheostomy on average 13.5% of patients died (drawn from 14 respondents from a population of 1687 intubated and ventilated patients).

With regards to tracheostomy technique and outcome, 3 out of 50 respondents gave preference to a percutaneous tracheostomy. An open surgical approach was favored by 8 out of 50 respondents. Other respondents (20/50) stated neither preference, with a remaining 19/50 unable to contribute.

## 4 DISCUSSION

The COVID‐19 pandemic has resulted in unparalleled demand on critical care services and the need for intubation and mechanical ventilation.^1^ Those who become critically ill do so due to rapid progression of pneumonia to a severe acute respiratory distress syndrome leading to respiratory failure and death.^3^, ^12^, ^13^ Data from Wuhan suggests the median time from hospital admission to death was 5 days, the mean 11 days.^14^

The results of this survey would suggest that approximately 1 out of every 10 intubated and ventilated patients require a tracheostomy. Furthermore, this intervention is of benefit with patients successfully weaned with only the minority dying following this intervention.

Current guidelines published by the American Academy of Otorhinolaryngology‐Head and Neck Surgery recommend that tracheostomy should not be performed prior to 14 days of intubation.^15^ The results of this survey would suggest that units are adopting a similar policy with few undertaking early tracheostomy routinely.

One should, however, be aware of the potential risks of prolonged ventilation in those who may be weaned. These include late tracheal ulceration, stenosis and tracheo‐esophageal fistula.^5^ Acquired critical care illness is also likely to become more prevalent.^16^ More patients will require prolonged ventilation and the muscular atrophy associated may prolong or prevent weaning.^17^ Anecdotal reports of glottic and supraglottic swelling and ulceration may also prohibit extubation and prolong the need for intubation, sedation and ventilation, that may be overcome by tracheostomy.

This survey failed to establish any clear benefit with regards to tracheostomy technique. Comments included by respondents generally explained that intervention was taken on a case by case basis and dependent on local surgical experience.

The results of this survey represent a snapshot of the current activity of several international units in a rapidly changing clinical environment. Limitations include the number of respondents with COVID positive patients concentrated in specific units. The management of this novel infection remains supportive with no clear evidence from recently initiated research trials.

During this time of stretched resources and limited personnel the authors are immensely grateful to those who have taken the time to respond to this survey to support the decision making of colleagues throughout the world.

## 5 CONCLUSIONS

This survey was undertaken to assess the current Otorhinolaryngology practice of tracheostomy intervention in COVID patients ventilated in Intensive Care Units.

While this survey represents a snap‐shot of current activity in a rapidly changing clinical environment, it does nonetheless suggest that there is a place for tracheostomy intervention in COVID‐19 positive intubated patients, but that this intervention is performed later (on average day 14) than would be classically recommended (days 7‐10).

There is no convincing data to support outcomes to be superior with either open surgical vs percutaneous approaches, and at present the decision depends on surgical experience and what is deemed to be safest for both patient and staff.
